# Conversion of Vertical Sleeve Gastrectomy to a Functional Single-Anastomosis Gastric Bypass: Technique and Preliminary Results Using a Non-Adjustable Ring Instead of Stapled Division

**DOI:** 10.1007/s11695-016-2392-9

**Published:** 2016-10-05

**Authors:** Francesco Greco

**Affiliations:** Head of Bariatric Surgery Unit, Clinica Castelli, Bergamo, Italy

**Keywords:** Sleeve gastrectomy, Single anastomosis, Mini-bypass, Re-do surgery, Fobi ring, Weight regain

## Abstract

**Background:**

Recent data show that some patients will have insufficient weight loss or experience weight regain after sleeve gastrectomy. Dilation of the sleeve over time or use of an inadequate technique may contribute to relapse of morbid obesity. Repeat sleeve gastrectomy is the most obvious option but requires stapling of scarred tissue, has a higher risk of leakage, and is prone to re-enlargement with time. We herein describe a simple and innovative technique with which to revise vertical sleeve gastrectomy (VSG) into functional single-anastomosis gastric bypass (f-SAGB).

**Materials and Methods:**

Twelve VSGs were converted to f-SAGB by placing a GaBP Ring (Bariatec Corp., Palos Verdes Peninsula, CA, USA) at the base of the “sleeve” and performing the anastomosis above the ring. The length of the biliopancreatic loop was chosen according to the volume of the pouch and the patient’s residual eating capability.

**Results:**

All procedures were completed by laparoscopy and were uneventful. The average decrease in the body mass index was from 41.0 to 29.5 kg/m^2^ at the 12-month follow-up. No ring-related complications were reported.

**Conclusion:**

f-SAGB is a low-risk and effective option with which to revise VSG in patients with inadequate weight loss. Avoiding detachment of the pouch from the antrum assures full reversibility of the procedure and preserves the chance to explore the remnant stomach and biliary tree.

## Introduction

Bariatric surgery is an evolving science, and the perfect surgical intervention does not exist. Although the effectiveness of bariatric surgery is no longer questionable, recurrence of obesity remains a major concern for bariatric patients. We have thus assisted in the decline in performance of adjustable gastric banding and the progressive disappearance of vertical-banded gastroplasty. The rising number of patients undergoing a second bariatric procedure has captured the attention of the bariatric surgery community, making revision surgery one of the main research topics in the recent years [1, 2].

Poor results after vertical sleeve gastrectomy (VSG) may be related to various factors, such as incorrect indications, patient behavior, and biology, or use of a poor operative technique (e.g., when the sleeve is too short, too large, or incomplete). Repeat sleeve gastrectomy is the most obvious option, but stapling of scarred tissue leads to an increased risk of leakage; additionally, the re-sleeved pouch is prone to re-enlargement with time [1, 3].

Another strategy involves revision of VSG into Roux-en-Y gastric bypass or a malabsorptive procedure [2, 4, 5]. The gastric sleeve itself, in its original description by Hess et al. [6], is the first step of biliopancreatic diversion with duodenal switch. Single-anastomosis duodenal switch was more recently proposed as an effective salvage procedure [7].

Single-anastomosis gastric bypass (SAGB) has gained popularity, and excellent results have been reported in terms of high rates of weight loss and weight maintenance and a very low rate of complications [8–13]. SAGB has good outcomes and has been proposed as a revision surgery after adjustable gastric banding or VSG because of its combined restrictive and malabsorptive effects [4, 5, 14, 15].

We herein describe the original functional SAGB (f-SAGB) technique as our revision strategy after VSG.

## Materials and Methods

Twelve patients (10 females, 2 males; age, 43 ± 8 years) who previously underwent VSG were referred to our unit for insufficient weight loss or weight regain. The average body mass index (BMI) at the time of VSG was 48.4 ± 12.4 kg/m^2^. After VSG, the average BMI decreased to 35.0 ± 14.3 kg/m^2^ and later increased to 41.0 ± 11.7 kg/m^2^ at the time of revision surgery. The mean delay from VSG to f-SAGB was 37 months (range, 13–60 months). Five patients had previously undergone gastric band removal; in five cases, the primary surgery was performed by laparotomy.

**Fig. 1:**
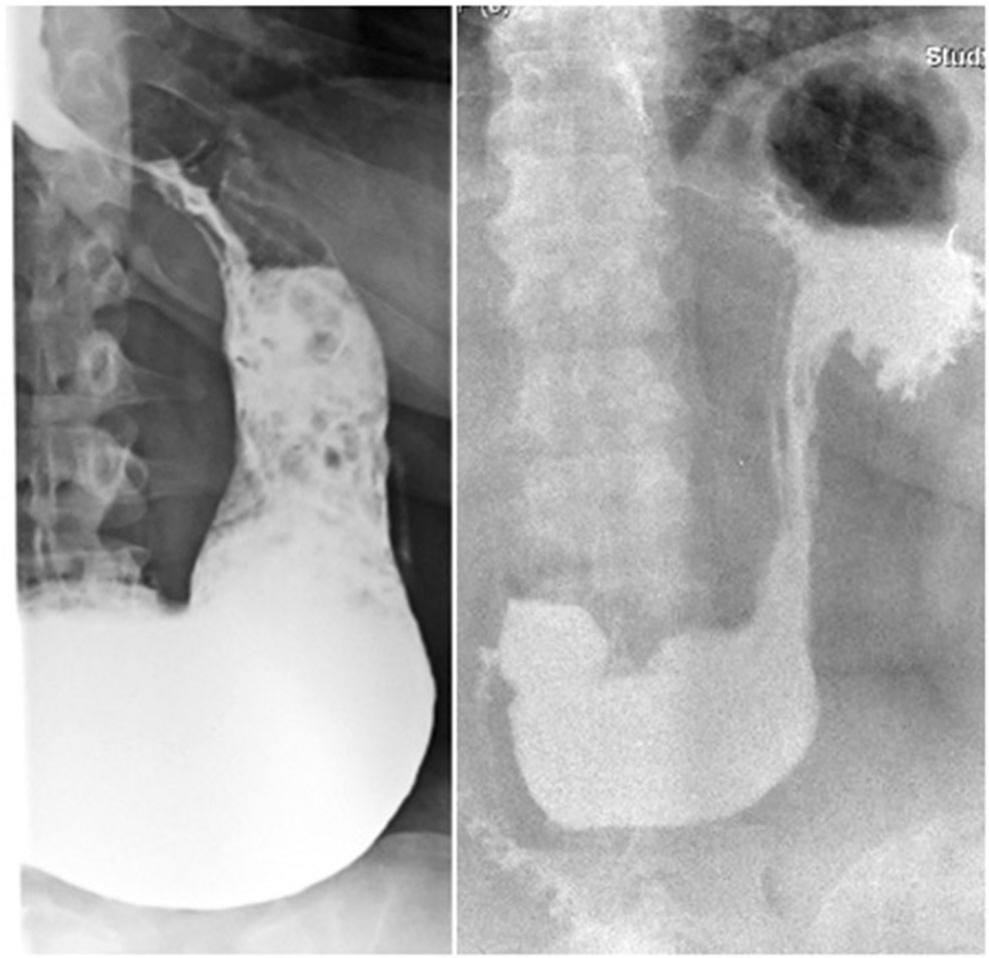
Group A (*left*): enlargement of the pouch; group B (*right*): dilated gastric fundus

According to the preoperative X-ray swallow, we classified the patients into two groups: those with a slight to moderate enlargement of the pouch (group A, *n* = 5) and those with a dilated gastric fundus (group B, *n* = 7) (Fig. 1). Eating habits were investigated to assess the degree of restriction for food and liquids: all patients in group B exhibited normal eating behavior, while mild restriction was reported by the patients in group A.

Each patient received a detailed description of the potential collateral effects and complications related to this surgery, and each patient provided written informed consent.

Cefazolin (2 g) was administered as prophylaxis. An antithrombotic agent (enoxaparin, 4000 IU/day) was administered 4 h after the end of the procedure and discontinued after 15 days.

The procedure was a three-trocar laparoscopy; the trocars were placed according to the patient’s anatomy, surgeon’s comfort, and previous scars. After establishment of pneumoperitoneum with a Veress needle in the left subcostal margin, access to the peritoneal cavity was gained with a 5-mm optic trocar in the epigastric region, about 15 to 20 cm from the xiphoid and 2 to 3 cm to the right of the midline. A 12-mm trocar was placed four to five fingers from the left subcostal margin at the level of the anterior axillary line; the last 5-mm trocar was placed near the right subcostal margin 3 to 4 cm from the midline, trespassing the falciform ligament. A 30-degree, 5-mm camera was positioned in the epigastric trocar and fixed to an autostatic holder (Karl Storz, Tuttlingen, Germany).

**Fig. 2:**
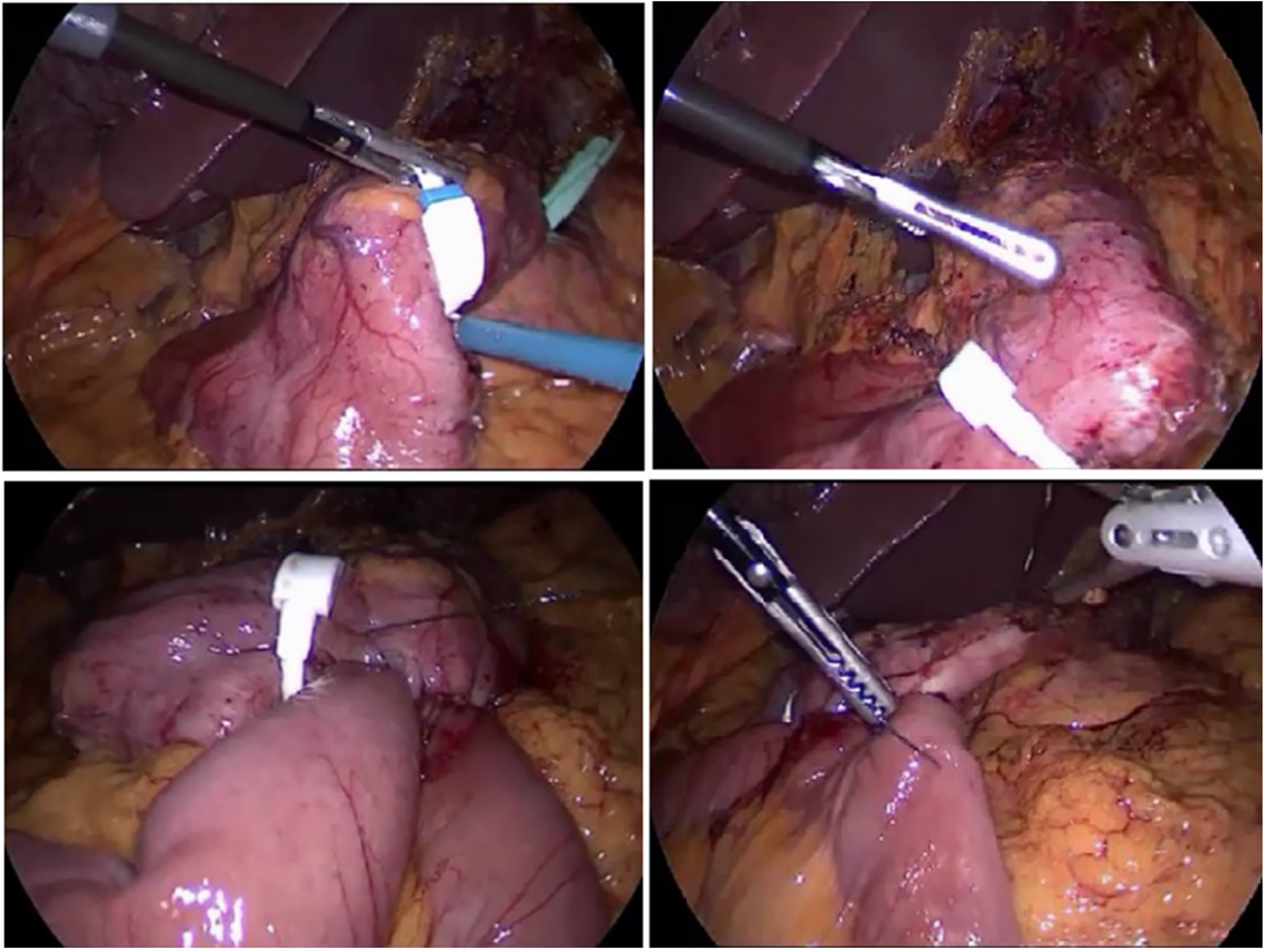
The f-SAGB technique involves placement of a non-adjustable ring at the base of the sleeve instead of stapling and dividing the pouch from the antrum

The surgeon stood on a 15-cm-high footboard on the right side of the patient. The patient was placed in the head-up position and rotated to the right side to approximate the superior pole of the spleen and the left 12-mm trocar to the surgeon. The gastric sleeve is usually involved in the formation of firm adhesions among the left lobe of the liver, the omentum, and the staple line; moreover, the esophagogastric junction is often involved in the formation of dense scar tissue in patients who have previously undergone gastric banding. A 40-Fr orogastric tube was moved forward, pushing the pouch and exposing the angular incisure. Dissection was confined to this portion of the gastric wall and extended about 3 to 5 cm on both sides of the lesser and greater curvatures to avoid damage at the esophagogastric junction and bleeding. A GaBP Ring (Bariatec Corp., Palos Verdes Peninsula, CA, USA) was then introduced into the peritoneal cavity and placed over the sleeve as distally as possible. The gastrotomy was opened just above the ring, 12 to 15 cm from the esophagogastric junction. No stitches were released to maintain the position of the ring (Fig. 2).

The second step of the procedure involved measurement of the bowel. The entire intestine was measured, and the length of the bypass was chosen according to the size of the pouch found at surgery and the patient-reported degree of restriction for food and liquids.

The patient was placed in a horizontal position with the surgeon standing at the left shoulder of the patient. The omentum was gently pulled upward, exposing the ileocecal valve (ICV). After marking a point 300 cm proximal to the ICV, an absorbable clip was released as a marker on both sides of the mesentery.

The surgeon again moved to the right side of the patient and began measuring from the ligament of Treitz. Measurement of the entire bowel was completed when the mark previously placed 300 cm from the ICV was reached.

The bowel was progressively moved upward during measurement, keeping the inferior pole of the spleen as a target. An enterotomy was made, and a 30-mm Tri-Staple Endo GIA (Covidien/Medtronic, Minneapolis, MN, USA) was introduced within the bowel and directed proximally. The bowel was rolled 270° with rotation on the major axis of the stapler to spread the tension on the intestinal wall. With a counterclockwise translation, the limb was approximated to the gastric pouch. While the patient was placed in the reverse Trendelenburg position, the pouch was pulled down; the ring was handled with the surgeon’s left hand. Laterolateral anastomosis was then performed. The service hole was closed with a two-layer barbed suture (V-Loc; ^Medtronic^).

**Fig. 3:**
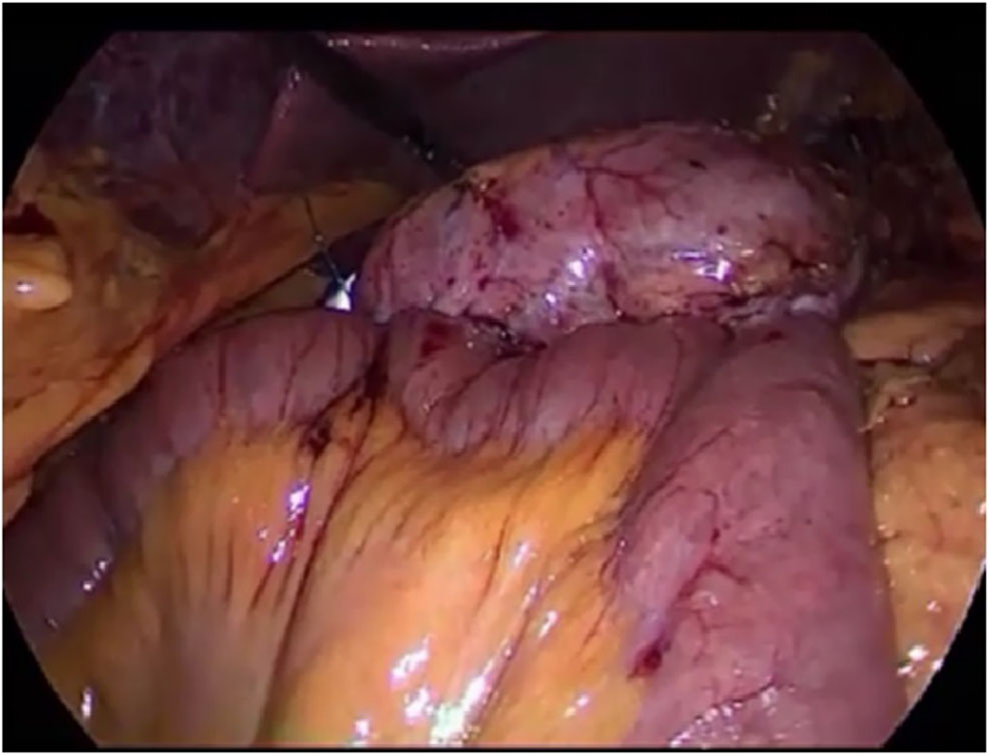
Methylene blue test at the end of the procedure demonstrate the transit through the anastomosis

The last step of the procedure involved a creation of the antireflux mechanism by suturing the afferent loop to the pouch for a length of 5 to 7 cm. A methylene blue test was performed to check the anastomosis site for leaks and confirm the preferential route of the gastric contents through the intestinal loop (Figs. 2 and 3).

## Results

**Fig. 4:**
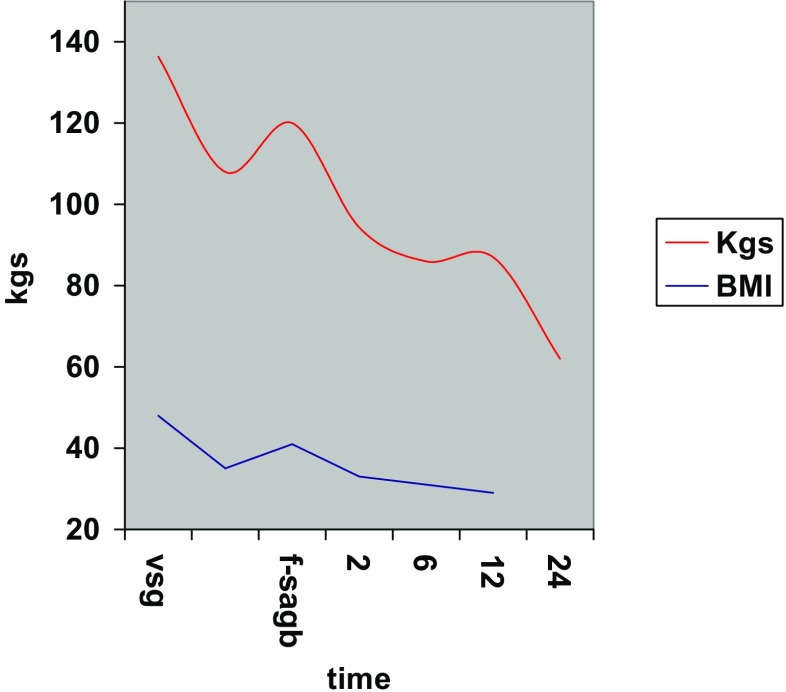
Weight/BMI chart. After conversion from VSG to f-SAGB average BMI decrease from 41 ± 11.7 to 29.5 ± 9 and average weight from 117 kg ± 40 to 84 kg ± 31.2 at 12 months follow-up

All procedures were completed by laparoscopy and were uneventful. A liquid diet was started on the first postoperative day. The postoperative hospital stay was <72 h in all cases. The average BMI had decreased from 41.0 ± 11.7 to 29.5 ± 9.0 kg/m^2^ at the 12-month follow-up. No ring-related complications were reported (Fig. 4).

## Discussion

Mini-gastric bypass is reportedly a relatively simple, rapid, and effective technique, the long-term results of which appear to be equal or better than those of standard Roux-en-Y gastric bypass (RYGB) [9–12, 16, 23]. A long lesser curvature conduit and long biliary limb explain the physiology of modified Billroth II reconstruction, which has a non-obstructive and balanced restrictive–malabsorptive effect [17, 18]. Excellent results of mini-gastric bypass as a revision surgery have been reported after restrictive procedures (adjustable gastric banding, sleeve gastrectomy) [14, 15].

The f-SAGB technique we have herein described involves placement of a non-adjustable ring (Fobi ring) at the base of the sleeve instead of stapling and dividing the pouch from the antrum. The ring is responsible for light compression of the gastric wall; the increased pressure above this neo-pylorus diverts food and liquids through the anastomosis into the bowel loop. The size of the ring is chosen according to the size of the pouch.

Prosthetic devices have been used in bariatric operations to control the outlet of the gastric pouch and thus maintain weight loss. A complication of these prostheses is erosion or migration into the gastric lumen. Fobi et al. [19] reported <2 % incidence of band erosion after transected banded vertical gastric bypass in patients presenting with symptoms of obstruction, pain, and bleeding.

Notably, when performing f-SAGB, the Billroth II gastroenterostomy creates a low-pressure system above the ring and facilitates transit through the anastomosis and not through the ring. Thus, we can imagine that the stress on the gastric wall at this level is minimal. Additionally, no new staple lines are in contact with the foreign body, and the integrity of the serosa is preserved because no sutures are released, reducing the risk of slippage and migration.

**Fig. 5:**
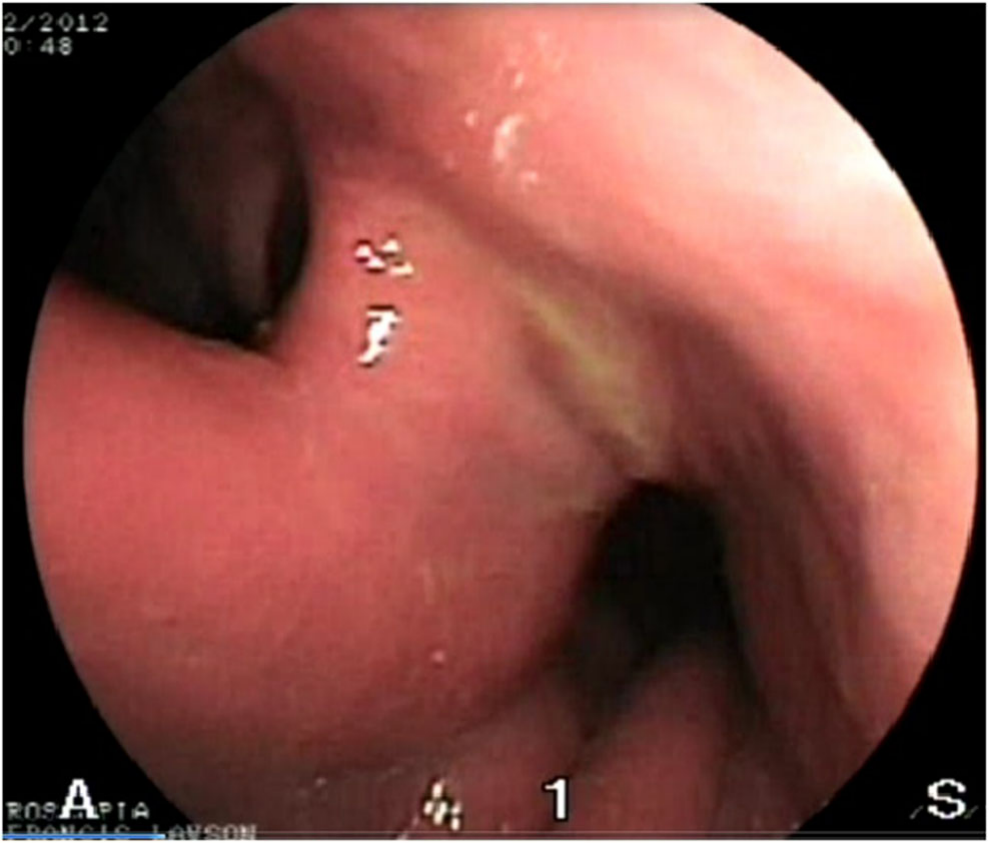
Fibroscopy demonstrate the laterolateral anastomosis (*left side*) and the neo-pylorus (right side). The preserved duodenal continuity in this f-SAGB technique allows for exploration of the remnant stomach and biliary tree

**Fig. 6:**
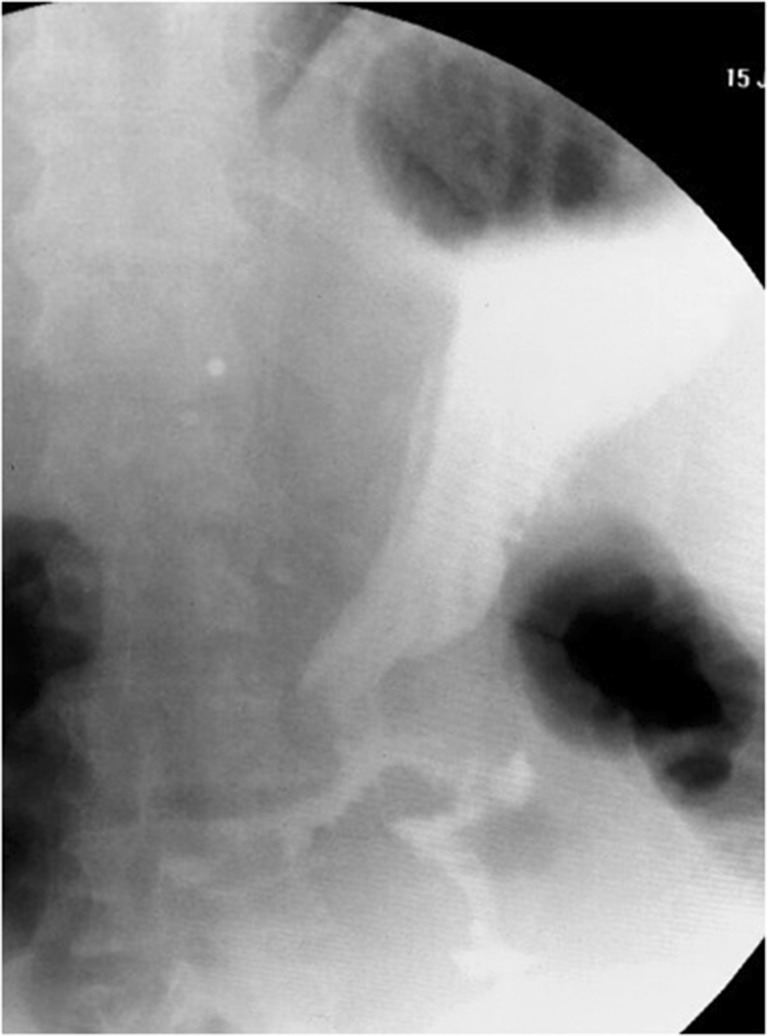
Postoperative X-ray swallow examination shows a preferential way of the contrast through the anastomosis

Furbetta [20] described the performance of functional RYGB by placing the anastomosis above the ring in adjustable gastric banding, and Amenta and Cariani [21] reported their results of functional RYGB on a vertical-banded gastroplasty. These authors’ experiences suggest that differential pressure above the ring is responsible for the transit of liquid and food in a preferential way through the anastomosis. This is exactly what we observe in postoperative X-ray swallow examinations, which shows minimal or no transit through the ring. Instead, fibroscopy reveals the laterolateral anastomosis, and the neo-pylorus that can be easily passed through (Figs. 5 and 6).

The preserved duodenal continuity in this f-SAGB technique allows for exploration of the remnant stomach and biliary tree in patients with malignancy, bleeding, or lithiasis. Reversibility is also assured and only requires cutting of the ring and division of the anastomosis.

We intentionally avoid re-sleeving the pouch to reduce the risk of leakage, and dissection involves only a limited portion of the gastric wall [1]. The length of the bypass is not standardized, and a larger pouch will result in a more distal bypass. Our strategy is to place the anastomosis 250 cm from the ligament of Treitz in patients with a slightly dilated sleeve and residual restriction for food (group A), while patients without any signs of restriction (group B) require the greatest malabsorptive effect; this is reached when the anastomosis is placed 300 cm from the ICV as we previously described for ileal food diversion (IFD) [22]. IFD is a non-restrictive single-anastomosis gastric bypass that creates a large gastric pouch (250–300 ml) with a 300-cm alimentary/common limb. This original configuration of the pouch of the IFD is similar to an incomplete VSG when the staple line is turned away from the angle of His, leaving in place a portion of the gastric fundus. This type of malabsorptive single-anastomosis gastric bypass functionally works like a biliopancreatic diversion.

## Conclusion

Revision surgery is technically demanding and may be associated with a high complication rate. The f-SAGB is our revision strategy after VSG. Re-sleeving of the pouch is avoided, and the anastomosis is placed above a non-adjustable ring without division of the pouch from the gastric antrum. The length of the bypass is tailored according to the size of the pouch to modulate the degree of malabsorption of the procedure. The access to the biliary tree and remnant stomach is preserved, and full reversibility is easy to obtain. Device-related complications must be considered but have not yet been reported. Although our preliminary results are encouraging, larger series and longer follow-ups are necessary.
